# High Bacterial Contamination Load of Self-Service Facilities in Sakaka City, Aljouf, Saudi Arabia, with Reduced Sensitivity to Some Antimicrobials

**DOI:** 10.3390/microorganisms11122937

**Published:** 2023-12-07

**Authors:** Ahmed E. Taha, Abdulbaqi S. Alduraywish, Ali A. Alanazi, Abdulaziz H. Alruwaili, Abdulaziz L. Alruwaili, Mmdoh M. Alrais, Abdulkareem A. Alyousef, Abdullah A. Alrais, Meshal A. Alanazi, Sultan N. Alhudaib, Bandar M. Alazmi

**Affiliations:** 1 Microbiology and Immunology Unit, Department of Pathology, College of Medicine, Jouf University, Sakaka 72388, Saudi Arabia; 2 College of Medicine, Jouf University, Sakaka 72388, Saudi Arabia

**Keywords:** antibacterial surfaces, bacterial transmission, contamination, drug resistant staphylococci, *Escherichia coli*, prevention, technology

## Abstract

Although self-service facilities (SSFs) have been used on a large scale worldwide, they can be easily contaminated by microorganisms from the hands of their sequential users. This research aimed to study the prevalence and antimicrobial susceptibility/resistance of bacteria contaminating SSFs in Sakaka, Aljouf, Saudi Arabia. We randomly swabbed the surfaces of 200 SSFs, then used the suitable culture media, standard microbiological methods, and the MicroScan WalkAway Microbiology System, including the identification/antimicrobial susceptibility testing-combo panels. A high SSFs’ bacterial contamination load was detected (78.00%). Ninety percent of the samples collected in the afternoon, during the maximum workload of the SSFs, yielded bacterial growth (*p* < 0.001 *). Most of the contaminated SSFs were supermarket payment machines, self-pumping equipment at gas stations (*p* = 0.004 *), online banking service machines (*p* = 0.026 *), and barcode scanners in supermarkets. In the antiseptic-deficient areas, 55.1% of the contaminated SSFs were detected (*p* = 0.008 *). Fifty percent of the contaminated SSFs were not decontaminated. The most common bacterial contaminants were *Escherichia coli* (70 isolates), *Klebsiella pneumoniae* (66 isolates), *Staphylococcus epidermidis* (34 isolates), methicillin-resistant *Staphylococcus aureus* (18 isolates), and methicillin-sensitive *Staphylococcus aureus* (14 isolates), representing 31.53%, 29.73%, 15.32%, 8.11%, and 6.31% of the isolates, respectively. Variable degrees of reduced sensitivity to some antimicrobials were detected among the bacterial isolates. The SSFs represent potential risks for the exchange of antimicrobial-resistant bacteria between the out-hospital environment and the hospitals through the hands of the public. As technology and science advance, there is an urgent need to deploy creative and automated techniques for decontaminating SSFs and make use of recent advancements in materials science for producing antibacterial surfaces.

## 1. Introduction

With the development of technology and science, self-service facilities (SSFs) have been used on a large scale all over the world [1]. Furthermore, with exponentially increasing numbers of severe acute respiratory syndrome coronavirus 2 (SARS-CoV-2) cases, the need for digital technology has grown considerably in a trial to limit the spread of this fatal virus [2].

Automatic teller machines (ATMs) and other online banking services, self-pumping at gas stations, supermarket barcode scanners, self-ticket purchasing on the internet, self-boarding passes at airports, and self-check-out at hotels and libraries are typical examples of self-service technologies. Touch screens can give users the false impression that they are microbial-free. The problem of the accumulation of microbiological threats on the surfaces of touch screens is well known to manufacturers [3].

The SSFs’ touch surfaces can be easily contaminated by microorganisms from the hands of the users and act as vehicles for transmitting pathogenic multi-antibiotic-resistant bacteria between the sequential users [4]. Furthermore, the contamination of touch surfaces can occur easily in humid and hot environments [5], as in many regions of Saudi Arabia [6]. 

Human skin provides an ideal habitat for the growth of many bacteria that can live on the skin as commensal microbiota. In a Saudi study aimed at characterizing the diversity of skin microbiota in the healthy Saudi population in Riyadh city, thirty-three bacterial species were isolated from males, while 24 species were identified from females. Micrococcus species, Pantoea species, *Enterobacter cloacae*, *Enterococcus casseliflavus*, *Klebsiella pneumoniae* (*K. pneumoniae*), *Kocuria kristinae*, *Serratia fonticola*, *Serratia odosifera*, *Sphingomonas paucimobilis*, *Staphylococcus* (*S.*) *gallinarum*, *S. hominis*, and *S. lentus* were recovered from the hands of the elderly, while Micrococcus species, *Sphingomonas paucimobilis*, *S. epidermidis*, *S. haemolyticus*, and *S. saprophyticus* were isolated from the hands of young males. *S. haemolyticus*, *S. warneri*, Micrococcus species, *S. epidermidis*, *S. xylosus*, *S. aureus*, and *Kocuria kristinae* were recovered from the hands of the elderly, while Micrococcus species, *S. epidermidis*, *S. warneri*, *S. hominis*, and *Pseudomonas aeruginosa* were isolated from the hands of young females. More bacterial isolates were found in the elderly than in the young [7].

Cave and his study team reviewed the literature for antimicrobial-resistant (AMR) bacteria detected on surfaces in many public places. The authors reported that Enterobacteriaceae and *Staphylococcus* species represent dangerous threats to human health. From the studies available to them, it seems many of the clinically important AMR isolates originate from hospitals and may be transmitted to the community via high-touch surfaces (HTS) of public transportation systems that patients and staff use to get to and from healthcare facilities or via hired bicycles shared by individuals with similar lifestyles [4]. 

Antimicrobial resistance is a worldwide crisis [8]. If significant worldwide action is not taken, it was predicted that by 2050, 10 million people might die yearly from AMR infectious pathogens, with a very high annual economic cost of USD 100 trillion [9]. With the emergence of the antimicrobial resistance problem, the bacteria contaminating SSFs can be challenging to eradicate, with fatal consequences, especially in immunocompromised people, including the elderly ones [1]. 

Antimicrobial resistance is not a recently generated phenomenon. It can arise through random mutations driven by selection due to antimicrobial pressure or other environmental stress factors, or through inter-species horizontal transfer of resistance genes including plasmids, transposons, pathogenicity islands, chromosomal cassettes, or prophages [10,11]. Resistance genes can then be wildly distributed in many other areas through the human movement or the food chain [12].

Public settings should not be ignored, even if most antimicrobial resistance surveillance studies to date have concentrated on healthcare settings in many countries [1,13,14,15,16,17]. Overall, very little is known about AMR bacteria that are found in public areas, including whether or not the resistance is waning or growing there. Furthermore, very little is known about AMR bacteria on hand-touched surfaces, even though most global investigations have focused on public transportation networks. In Saudi Arabia, a greater comprehension of the possible role of SSFs as bacterial reservoirs is necessary to assist health policy makers in developing the best preventative and control strategies, which may include using modern decontamination techniques. As far as we know, there is no previous study that directly tested the present hypothesis in Saudi Arabia. The aim of this research is to study the prevalence of bacterial contamination load in SSFs as potential sources for bacterial pathogen transmission in Sakaka, Aljouf, Saudi Arabia, and the antimicrobial susceptibilities/resistances profiles of the isolates.

## 2. Materials and Methods

### 2.1. Study Design, Data, and Sample Collection

The local committee of bioethics (LCBE) of Jouf University in Saudi Arabia granted bioethical approval (number 22-10-43). For the total of about 400 SSFs in Sakaka city, Aljouf, Saudi Arabia, the sample size was determined using an online (Raosoft) sample size calculator (http://www.Raosoft.com/samplesize.html; accessed on 28 September 2021) with a margin of error of 5%, response distribution of 50%, and confidence level of 95%.

A cross-sectional study was performed to randomly swab 200 SSFs in Sakaka city, Aljouf, Saudi Arabia, after obtaining the required permissions. The sequential inclusion of SSFs in the study continued randomly until the calculated sample size was achieved. Flipping a coin is a simple procedure that was used to ensure randomization during sample collection. Data for each SSF were collected regarding its type, decontamination (cleaning and disinfection), the presence of breaks in its screen, and the constant availability of hand antiseptics near the SSF, as shown in Table 1 and Table 2. The privacy and confidentiality of the owners of the sampling sites were protected.

Sterile cotton-tipped swabs with amies transport media (GlobalRoll^®^, Hangzhou, China) were used. We swabbed the 200 SSF, 100 of them in the early morning before working, and the other 100 in the afternoon during the maximum workload. The sterile swabs moistened with the transport media were rolled over all exposed outer surfaces of the SSF. During swabbing, we focused on the areas that are most commonly in contact with the tips of fingers, such as buttons and touch screens. Each sample was collected in a sterile bag and carried in an icebox to the Microbiology and Immunology Laboratory at Jouf University’s College of Medicine for further processing. 

### 2.2. Bacterial Isolation and Identification

On arrival, the samples were processed using aseptic techniques to avoid contamination. All media included in this study were prepared according to the manufacturer’s instructions. The swabs were put in tubes containing five ml of double-strength brain–heart infusion (BHI) broth (Oxoid, Hampshire, UK) and incubated aerobically for 24 h at 37 °C. The swabs were inoculated on nutrient, blood, and MacConkey agar plates (Oxoid, Hampshire, UK) and incubated aerobically for 24–48 h at 37 °C. After incubation, colonies were examined by Gram-stained films. To obtain a pure single colony of each bacterial isolate, the isolates were grown on nutrient, blood, and MacConkey agar plates and incubated aerobically at 37 °C for 24 h.

The colonies with typical characteristics of staphylococci (Gram-positive, cluster-forming, non-spore-forming, facultative anaerobe, growing on blood and nutrient agar) were subcultured on Mannitol salt agar [18] and examined by catalase (they were catalase positive) and coagulase tests [19].

### 2.3. Bacterial Identification, Confirmation, and Antimicrobial Susceptibility Testing (AST)

Automated AST systems are widely used in clinical laboratories and have numerous advantages, such as ease of use, data management with expert system analysis, reduced sample handling times, and reproducibility [20,21]. MicroScan systems have provided gold-standard bacterial identification and susceptibility products, confronting emerging resistance with speed and accuracy [22].

The pure colonies of the isolates were identified by standard microbiological methods, including Gram stain, size, color, and shape. The isolates were classified into Gram-negative, and Gram-positive groups. Confirmation of isolates and their AST was conducted using the automated identification (ID)/AST MicroScan WalkAway Microbiology System (DxM 1096; Beckman Coulter, Inc., Sacramento, CA, USA) for rapid and accurate reporting. We used the ID/AST-combo Gram-negative breakpoint (BP) panel (Neg BP Combo 50; MicroScan catalog number B1016-189) and Gram-positive panel (Pos Combo 31; MicroScan catalog number B1016-139) that adhere to the guidelines established by the Clinical and Laboratory Standards Institute (CLSI) [23].

The quality control (QC) was achieved by using ATCC strains (*Escherichia coli*; *E. coli*; ATCC10536; *K. pneumoniae*; ATCC10031; and *S. aureus*; ATCC25923) as positive controls. QC testing was performed in triplicate. Triplicate testing was conducted for each isolate. The negative controls of the study during bacterial culture were non-inoculated blood and MacConkey agar plates incubated aerobically for 48 h at 37 °C. All data were interpreted according to the CLSI [23].

### 2.4. Data Analysis

Data were fed to the computer and analyzed using IBM SPSS software package version 20.0. (Armonk, NY, USA: IBM Corp.) Categorical data were represented as numbers and percentages. A Chi-square test was applied to investigate the association between the categorical variables. Alternatively, the Fisher Exact correction and Monte Carlo correction tests were applied when more than 20% of the cells had an expected count less than 5. The significance of the obtained results was judged at the 5% level.

## 3. Results

The prevalence of bacterial contamination of SSFs in Sakaka city, Aljouf, Saudi Arabia, was screened in 200 samples. We swabbed 100 SSF in the early morning before working, and the other 100 in the afternoon during the maximum workload. Samples were processed and cultured on appropriate media under suitable incubation conditions. No bacterial growth was detected in 44 samples after 48 h of incubation (22.00%; *n* = 44/200). Bacterial contamination was detected in 156 samples. Thus, the high bacterial contamination load of the SSFs was 78.00%; *n* = 156/200.

Table 1 and Table 2 compare the effects of time of sample collection, type of SSF, presence of hand antiseptics near the SSF, presence of breaks in the screen of SSF, and SSF decontamination on the extent of their bacterial contamination load. We observed a highly significant association (*p* < 0.001 *) between SSFs’ bacterial contamination and the time of sample collection (90.0% of the samples collected in the afternoon, during the maximum workload of the SSFs, yielded bacterial growth). Most of the contaminated SSFs were supermarkets’ payment machines, self-pumping at gas stations (*p* = 0.004 *), online banking services (*p* = 0.026 *), and barcode scanners in supermarkets.

Another statistically significant association (*p* = 0.008 *) was detected between SSFs’ bacterial contamination and the absence of hand antiseptics in the nearby area (55.1% of the contaminated SSFs were detected in hand antiseptic-deficient areas). The data show that 50.0% of the contaminated SSFs were not decontaminated; nevertheless, a statistically significant association was not detected between SSF decontamination and their positivity for bacterial growth (*p* = 1.000), as shown in Table 2.

**Table 1:** Comparison of the effect of time of sample collection, type of SSF, presence of hand antiseptics near the SSF, and presence of breaks in the screen of SSF on the extent of their bacterial contamination load (Total *n* = 200). Data shown are frequencies; *n* (%).

Bacterial Contamination Load of Self-Service Facilities	Total (*n* = 200)	Result		χ^2^	*p*
		Non-Contaminated *n* = 44 (22%)	Contaminated *n* = 156 (78%)		
**Time of sample collection**					
Early morning (before work)	100 (50%)	34 (77.3%)	66 (42.3%)	16.783 *	<0.001 *
Afternoon (After work)	100 (50%)	10 (22.7%)	90 (57.7%)		
**Types of self-service facilities**					
Online banking service machine	40 (20%)	14 (31.8%)	26 (16.7%)	4.924 *	0.026 *
Self-pumping equipment at the gas station	52 (25%)	4 (9.1%)	48 (30.8%)	8.383 *	0.004 *
Payment machine in the supermarket	56 (28%)	12 (27.3%)	44 (28.2%)	0.015	0.903
Supermarket barcode scanner	24 (12%)	4 (9.1%)	20 (12.8%)	0.452	0.501
Self-ticket purchasing at the airport	4 (2%)	0 (0%)	4 (2.6%)	1.151	^FE^*p* = 0.578
Self-boarding pass at the airport	4 (2%)	2 (4.5%)	2 (1.3%)	1.865	^FE^*p* = 0.211
Self-ticket purchasing at the train station	4 (2%)	2 (4.5%)	2 (1.3%)	1.865	^FE^*p* = 0.211
Self-boarding pass at the train station	4 (2%)	4 (9.1%)	0 (0%)	1.415	^FE^*p* = 0.577
Self-check-out at the hotel	4 (2%)	0 (0.0%)	4 (2.6%)	1.151	^FE^*p* = 0.578
Self-check-out at the library	8 (4%)	2 (4.5%)	6 (3.8%)	0.044	^FE^*p* = 1.000
**The constant presence of hand antiseptics near the SSF**					
No	120 (60%)	34 (77.3%)	86 (55.1%)	7.012 *	0.008 *
Yes	80 (40%)	10 (22.7%)	70 (44.9%)		
**Breaks in the screen of SSF**					
No	190 (95%)	42 (95.5%)	148 (94.9%)	0.025	^FE^*p* = 1.000
Yes	10 (5%)	2 (4.5%)	8 (5.1%)		

χ^2^: Chi square test. FE: Fisher Exact. *p*: *p* value for comparing between non-contaminated and contaminated. *: Statistically significant at *p* ≤ 0.05.

**Table 2:** Comparison of the effect of SSF decontamination on the extent of their bacterial contamination load (Total *n* = 200). Data shown are frequencies; *n* (%).

Bacterial Contamination Load of Self-Service Facilities	Total (*n* =200)	Result		χ^2^	*p*
		Non-Contaminated *n* = 44 (22%)	Contaminated *n* = 156 (78%)		
**SSF decontamination**					
No	100 (50%)	22 (50%)	78 (50%)	0.0	1.000
Yes	100 (50%)	22 (50%)	78 (50%)		
**-Frequency**					
*n*	100 (50%)	22 (50%)	78 (50%)	11.219	^MC^*p* = 0.061
Once a day	32 (16%)	6 (13.6%)	26 (16.7%)		
Twice a day	24 (12%)	2 (4.5%)	22 (14.1%)		
Once every 2 days	12 (6%)	2 (4.5%)	10 (6.4%)		
Once a week	12 (6%)	2 (4.5%)	10 (6.4%)		
Once a month	16 (8%)	8 (18.2%)	8 (5.1%)		
Once a year	4 (2%)	2 (4.5%)	2 (1.3%)		
**-Duration since last time SSF decontamination**					
*n*	100 (50%)	22 (50%)	78 (50%)	4.082	^MC^*p* = 0.529
<3 h	24 (12%)	6 (13.6%)	18 (11.5%)		
3–6 h	4 (2%)	2 (4.5%)	2 (1.3%)		
6–12 h	12 (6%)	2 (4.5%)	10 (6.4%)		
12–18 h	0 (0%)	0 (0%)	0 (0%)		
18–24 h	20 (10%)	2 (4.5%)	18 (11.5%)		
>24 h	40 (20%)	10 (22.7%)	30 (19.2%)		
**-Method of SSF decontamination**					
*n*	100 (50%)	22 (50%)	78 (50%)	9.350 *	0.025 *
Tissue	36 (18%)	14 (31.8%)	22 (14.1%)		
Water	32 (16%)	4 (9.1%)	28 (17.9%)		
Alcohol	32 (16%)	4 (9.1%)	28 (17.9%)		

χ^2^: Chi square test. MC: Monte Carlo. *p*: *p* value for comparing between non-contaminated and contaminated. *: Statistically significant at *p* ≤ 0.05.

**Figure 1:**
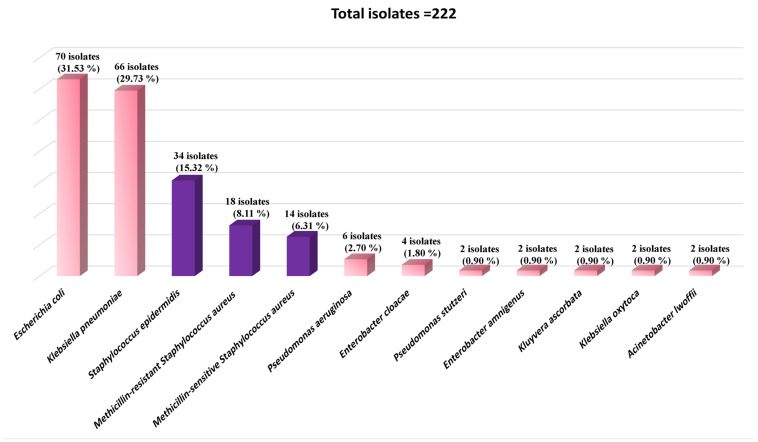
Results of isolate identification by the MicroScan Microbiology System. The data shown are frequencies, *n* (%). No bacterial growth was detected in 44 samples after 48 h of incubation (22.00%; *n* = 44/200).

Among the bacterial growth-positive samples, 90 swabs yielded a single bacterial organism (45.00%; *n* = 90/200), whereas 66 swabs yielded 2 bacterial organisms (33.00%; *n* = 66/200). Consequently, the total number of bacterial isolates was 222. The pure colonies of the isolates were identified by the standard microbiological methods and classified into Gram-negative and Gram-positive groups, then confirmed by the MicroScan Microbiology System. The Gram-negative isolates represented 70.27%; *n* = 156/222. The Gram-positive isolates represented 29.73%; *n* = 66/222. The most common bacterial contaminants were *E. coli* (70 isolates), *K. pneumoniae* (66 isolates), *S. epidermidis* (34 isolates), methicillin-resistant *S. aureus* (MRSA; 18 isolates), and methicillin-sensitive *S. aureus* (MSSA; 14 isolates) at frequencies of 31.53%, 29.73%, 15.32%, 8.11%, and 6.31%, respectively, as shown in Figure 1. 

The sensitivity, intermediate susceptibility, and resistance of the isolates to the tested antibiotics were detected by the MicroScan Microbiology System. Variable degrees of reduced sensitivity to some antibiotics were detected, as shown in Table 3 and Table 4.

## 4. Discussion

The problem of transmitting microbiological threats through smart devices is a general problem that affects all people, especially during and after the COVID-19 pandemic, which increased trust in technology, the popularity of touch screens, and direct human–machine interfaces (HMI) [3]. The exchange of microorganisms during the HMI is bidirectional between the surface being touched and the human skin (fingers/hands) [24]. Globally, hospital environmental screenings revealed that contaminated hospital environmental HTS are significant sources of microbial pathogens causing healthcare-associated infections (HAIs) because numerous microorganisms can survive on surfaces for hours to months, resulting in increased costs, morbidity, length of hospital stay, and mortality. Furthermore, enhanced cleaning and disinfection of such surfaces aid in the reduction in HAIs [13,16,17,25,26].

The extent to which such microbial contamination of other HTS outside the hospitals occurs remains undetermined. In this perspective, we aimed to determine the prevalence of the bacterial contamination load of SSFs as potential sources for bacterial pathogen transmission in Sakaka City, Aljouf, Saudi Arabia, and study the antimicrobial susceptibilities/resistances profiles of the isolates. 

In the current study, a high bacterial contamination load of the SSFs was detected (78.00%) in Sakaka City. A lower contamination rate (30.1%) was reported by Stepanovic et al. when they investigated the bacterial contamination load of the public transport system (trams, trolleybuses, and buses) in an urban community in Belgrade, Serbia [27]. A higher contamination rate (95.0%) was reported by Otter and French when they investigated the bacterial contamination of HTS in a public transport system (including trains, buses, stations, and phone boxes), a hotel, a museum, and a public area of a hospital in London, United Kingdom [28]. The results vary depending on the study location. The high bacterial contamination load of the SSFs detected in our research can be explained by the presence of 55.1% of the contaminated SSFs in hand antiseptics-deficient areas (*p* = 0.008 *; as shown in Table 1), the non-decontamination of 50.0% of the SSFs in Sakaka City, and the non-sufficiency of the decontamination of the other 50.0% of the SSFs regarding the frequency of decontamination, duration since last time SSF decontamination, and method of decontamination (*p* = 0.025 *) as shown in Table 2. 

In our research, a highly significant association (*p* < 0.001 *) was observed between SSFs’ bacterial contamination and the time of sample collection (90.0% of the sample collected in the afternoon, during the maximum workload of the SSFs, yielded bacterial growth). Similarly, some studies focused on the change in microbial contamination load before and after work and showed significantly increased microbial contamination at the end of the working day [1,29,30]. Wu and his colleagues reported more detection of microbial contaminants on HTS of the outpatient SSFs in the swabs collected after work than those collected before it [1]. Reynolds and his research team found that the HTS’ contamination levels could reach their maximum after only two hours of starting the work at an outpatient clinic [29]. 

Surfaces that are touched multiple times a day (particularly if this is by people with poor hand hygiene) and are not decontaminated regularly could facilitate the AMR bacterial transmission within the population [31]. The advent of COVID-19 has forced people to increase their dependence on using SSFs during routine daily activities. It was clear from the results of our study that most of the contaminated SSFs were supermarket payment machines, self-pumping equipment at gas stations (*p* = 0.004 *), online banking service machines (*p* = 0.026 *), and barcode scanners in supermarkets.

In the study, the Gram-negative isolates represented 70.27%. The Gram-positive isolates represented 29.73 percent. The most common bacterial contaminants were *E. coli*, *K. pneumoniae*, *S. epidermidis*, MRSA, and MSSA isolates at frequencies of 31.53%, 29.73%, 15.32%, 8.11%, and 6.31%, respectively.

Enterobacteriaceae are a group of bacteria, including *E. coli* and Klebsiella species, usually associated with the gut that can contaminate water, food, and environmental surfaces [32]. The presence of these bacteria is commonly associated with fecal contamination [33]. *E. coli* is one of the most well-studied bacterial species of the human gut microbiome [34]. *E. coli* populations can survive and even grow in open environments. Some *E. coli* strains can produce filamentous structures that extend from their cell surface and help them attach to surfaces [35]. 

Even though *K. pneumoniae* bacteria are widespread colonizers of the human gastrointestinal tract (GIT), skin, and throat, they can cause GIT infections, wound infections, sepsis, and pneumonia, particularly in immunocompromised individuals [36]. There have been growing concerns regarding *K. pneumoniae*, mainly due to their extensive β-lactamase production. *K. pneumoniae* may survive for ≥30 months in the environment [37]. Some studies detected Enterobacteriaceae bacteria in public areas such as public transportation systems [38], public-hired bicycles [39] in China, and public buses in Ethiopia [40].

Staphylococci can contaminate and live on inanimate objects for days to months [41], and these contaminated surfaces may have a role in this bacterial transmission [42]. Although coagulase-negative staphylococci (CoNS) are part of the core composition of the human’s skin and mucous membrane microbiota [43], they are also opportunistic bacteria that can persist and multiply on a wide variety of environmental surfaces, from community to hospital environments [44]. Many studies detected CoNS in non-healthcare public settings such as public restrooms [45], train stations [46], the metro system [47], university campuses [48], supermarkets, hotel rooms, restaurants, public libraries, the public transport system [49], and shopping centers [50].

Multi-drug-resistant MRSA was detected in many countries [51,52,53], including Saudi Arabia [54,55]. In a Saudi report, the total estimated prevalence of MRSA was 35.6%, with wide variations among different Saudi regions [56]. Saudi Arabia can be considered a hot spot for AMR bacteria such as MRSA because about 20% of its population are expatriates and because of the mass gathering of about four million Muslims during Hajj and Umra seasons [57]. The environmental (non-clinical) reservoirs of MRSA and the increase in MRSA infection in the non-clinical environment pose a serious public health concern [58]. MRSA isolates from non-hospital settings may have originated in healthcare facilities, or they may be de novo strains that developed after acquiring the *mecA* gene from methicillin-resistant bacteria [27]. MRSA isolates that originated in community settings have been reported to spread into hospitals [59].

Sexton and his research team reported that MRSA was isolated from every bus, subway, airplane, train, public toilet, and public office sampled in a study from Arizona and explained that the higher prevalence of CA-MRSA in the United States [60]. On the other hand, Stepanovic and his colleagues reported that the collected samples in their study were positive for methicillin-resistant coagulase-negative staphylococci (MRCoNS), but none for MRSA [27]. Furthermore, Otter and French detected MSSA but no MRSA in their above-mentioned study and explained that by the low prevalence of CA-MRSA carriage in the healthy population of London [28].

In the current study, reduced sensitivity to some antibiotics was detected with variable degrees, as shown in Table 3 and Table 4. Numerous studies reported AMR bacterial transmission to humans in many public settings, such as buses in Portugal [61], university classrooms in China [62], shopping baskets’ handles in Japan [63], subway trains in the United States [64], and railway stations in China [65]. 

The multi-drug-resistant nature of many Enterobacteriaceae pathogens could result in their difficult treatment [66,67]. In clinical settings, carbapenem and colistin antibiotics are the last resort to combat these bacteria [68]. Extended-spectrum -lactamase (*ESBL*) and mobile colistin resistance (*mcr*) genes have been found in carbapenem-resistant Enterobacteriaceae (CRE), which raises the risk of antimicrobial resistance spreading to other bacterial strains and makes many multidrug-resistant Enterobacteriaceae potentially untreatable [69,70,71,72]. In China, antibiotic-resistant Enterobacteriaceae were detected in higher rates in community settings near hospitals, which necessitates improved hygienic interventions in such areas [38,39].

The detection of *S. epidermidis* bacteria that are resistant/intermediately sensitive to cefoxitin and/or oxacillin is alarming, as they could represent sources for *mecA* gene transmission to *S. aureus,* resulting in increasing MRSA rates in the community. Transmission of the *mecA* gene to *S. aureus* from MRCoNS has been reported [73,74]. Similarly, Stepanovic and his colleagues found that approximately 50% of MRCoNS isolates exhibited resistance to beta-lactams and ≥2 other classes of antimicrobials [27]. In addition, Linezolid-resistant *S. epidermidis* was reported [75]. In contrast, in Turkish research, all MRSA isolates were susceptible to the tested antibiotics [76].

The surfaces in overcrowded public settings are recurrently touched by several people, and they can serve as a vector for the spread of AMR bacteria from person to person if people encounter them directly or indirectly through the shedding of bacteria from one person’s skin onto the surface, which then spreads to others who come into contact with the same surface [61,77,78].

As early as 1972, Spaulding classified inanimate surfaces into three categories (critical, semi-critical, and non-critical) according to the expected infection transmission risk [79]. HTS are considered non-critical items because they contact intact skin (but not mucous membranes), which represents a barrier against most pathogens. Thus, HTS could be decontaminated through cleaning followed by disinfection without the absolute need for sterilization [80].

The Healthcare Infection Control Practices Advisory Committee (HICPAC) endorsed cleaning followed by low- or intermediate-level disinfection for non-critical items, depending on the degree and nature of the contamination. For small surfaces, ethyl or isopropyl alcohol (60–90% *v*/*v*) may be used [81]. Alcohol is not suitable for large surfaces due to its rapid evaporation; thus, the contact time will be inadequate [82]. Triclosan is a broad-spectrum antimicrobial agent that is widely used for cleaning purposes in the form of soaps, detergents, or disinfectants. It has a wide activity range against several antibiotic-resistant bacteria, including *E. coli* and *S. aureus* [83].

Manual and automated techniques can be used for decontaminating HTS. The manual techniques include wiping or washing clothes with detergents or disinfectants. Manual cleaning is a critical step in the decontamination process because the physical removal of soil is required because its presence will impede the antimicrobial effects of the disinfectants, if necessary. Daily cleaning with neutral detergent wipes is usually sufficient to control the bioburden [31]. As a consequence of the environmental persistence and easy transmission of many microbial contaminants, optimal hand hygiene and chlorine-based disinfection of many surfaces are of major importance [84,85]. Recently, the novel and automated methods used for decontamination of HTS may include hydrogen peroxide, steam vapor, ozone, UV light, and high-intensity narrow-spectrum light [31].

With the development of technology and science, focusing solely on decontamination to reduce the bioburden on HTS does not become the only option. There are additional new strategies to prevent HTS from acting as microbial reservoirs, such as reducing the number of edges of touch screens available in public places to decrease the accumulation of microbiological threats and let them quickly decontaminate [3].

Recent breakthroughs in materials science have resulted in high-performance, multifunctional materials with bioactive characteristics. There are three basic approaches to developing antibacterial surfaces. The first is antibacterial agent release, which involves the release (through diffusion) of integrated antibacterial agents to deliver antibacterial action just where it is required. The second is contact-killing coating by enzymes or cationic chemicals (quaternary ammonium compounds, chitosan, antimicrobial peptides, etc.) that are covalently bonded to the material surface by flexible, hydrophobic polymeric chains to destroy the bacterial cell membrane upon contact. The third type is anti-adhesion coatings, which use bacterial repelling chemicals to prevent the first stage of bacterial biofilm development utilizing non-cytotoxic processes [86]. Many studies reported the possibility of microbial adhesion inhibition by repellent antimicrobial coats of copper, silver, zinc, bacteriophages, triclosan, polycations, or, even, light-activated radicals. However, the cost-effectiveness of new materials and technologies must be considered [31,86,87,88,89].

Although decorating metal complexes with metallic nanoparticles can improve metal complexes in different applications, including the biological field, with the advantages of their antimicrobial activities [90], concerns regarding durability, toxicity, resistance, and cost-effectiveness may be precluding a much wider application of the novel and automated methods for decontamination [31]. A limitation of our study was that we did not assess the survival time of the isolates on the surfaces of SSFs.

## 5. Conclusions

With the development of science and technology, more and more SSFs will be used, especially after the COVID-19 pandemic. As far as we know, this is the first report on the role of SSFs as potential sources of AMR bacteria in Sakaka City, Aljouf, Saudi Arabia, that could be valuable for health policy makers in improving the implemented infection control measures in the community. The results of the present study expand our knowledge of the presence of bacterial threats on the SSFs’ surfaces. A high bacterial contamination load of the SSFs detected in our research can be explained by the presence of most of the contaminated SSFs in hand antiseptic-deficient areas, the non-decontamination of many SSFs at all, and the defective decontamination of many SSFs regarding the frequency, method, and duration of SSF decontamination since last time.

SSFs act as bacterial reservoirs. As the transfer of bacterial threats between the community and the hospital apparently runs in both directions, bacterial contaminants within our immediate everyday environment increase the risk of transmission of these threats into hospital settings and vice versa. With the emergence of the antimicrobial resistance problem, the bacteria contaminating SSFs can be challenging to eradicate. All these data support the urgent need for prioritizing decontamination of SSFs to block the pathogen’s transmission cycle within public and hospital settings. Efficient manual cleaning at regular intervals is a very important initial step in the decontamination process. A reasonable and qualified SSFs decontamination plan can include many manual and automated techniques. Currently, these manual and automated techniques exhibit variable success.

Considering HTS in hospitals as models, we recommend cleaning followed by low- or intermediate-level disinfection for out-hospital SSFs with education of cleaning staff, their follow-up by checklists, and assessment of cleaning adequacy by direct observation, swab cultures, or agar slide cultures with feedback to the cleaning staff to improve the frequency of adequate decontamination. Cost-effectiveness studies are required for more investigation of automated methods and modern technologies used for decontamination of HTS to face the challenge of the worldwide spread of AMR microorganisms. 

## Figures and Tables

**Table 3:** Antibiotic resistance/susceptibility patterns of Gram-negative isolates (*n* = 156) by the MicroScan Microbiology System. Data shown are frequencies; *n* (%).

Antibiotics *		*Escherichia coli* *n* = 70 (44.87%)			*Klebsiella pneumonia* *n* = 66 (42.31%)			Other Gram-Negative Isolates *n* = 20 (12.82%)		
		R	I	S	R	I	S	R	I	S
Amikacin	*n*	0	0	70	0	0	66	0	0	20
	%	0.0	0.0	100.0	0.0	0.0	100.0	0.0	0.0	100.0
Amoxicillin/K Clavulanate	*n*	0	0	70	0	0	66	0	0	20
	%	0.0	0.0	100.0	0.0	0.0	100.0	0.0	0.0	100.0
Ampicillin	*n*	12	0	58	20	0	46	4	2	14
	%	17.1	0.0	82.9	30.3	0.0	69.7	20.0	10.0	70.0
Ampicillin/Sulbactam	*n*	0	0	70	0	0	66	0	0	20
	%	0.0	0.0	100.0	0.0	0.0	100.0	0.0	0.0	100.0
Aztreonam	*n*	4	2	64	4	0	62	4	2	14
	%	5.7	2.9	91.4	6.1	0.0	93.9	20.0	10.0	70.0
Cefazolin	*n*	8	0	62	12	2	52	6	4	10
	%	11.4	0.0	88.6	18.2	3.0	78.8	30.0	20.0	50.0
Cefepime	*n*	0	2	68	4	0	62	0	0	20
	%	0.0	2.9	97.1	6.1	0.0	93.9	0.0	0.0	100.0
Cefotaxime	*n*	2	8	60	4	2	60	4	2	14
	%	2.9	11.4	85.7	6.1	3.0	90.9	20.0	10.0	70.0
ESβL Confirmation: Cefotaxime/K Clavulanate	*n*	0	0	70	0	0	66	0	0	20
	%	0.0	0.0	100.0	0.0	0.0	100.0	0.0	0.0	100.0
Cefoxitin	*n*	0	0	70	0	0	66	0	0	20
	%	0.0	0.0	100.0	0.0	0.0	100.0	0.0	0.0	100.0
Ceftazidime	*n*	2	16	52	0	2	64	0	2	18
	%	2.9	22.9	74.3	0.0	3.0	97.0	0.0	10.0	90.0
ESβL Confirmation: Ceftazidime/K Clavulanate	*n*	0	0	70	0	0	66	0	0	20
	%	0.0	0.0	100.0	0.0	0.0	100.0	0.0	0.0	100.0
Cefuroxime	*n*	44	4	22	32	8	26	2	2	16
	%	62.9	5.7	31.4	48.5	12.1	39.4	10.0	10.0	80.0
Ciprofloxacin	*n*	0	8	62	0	4	62	0	0	20
	%	0.0	11.4	88.6	0.0	6.1	93.9	0.0	0.0	100.0
Colistin	*n*	0	0	70	0	0	66	0	0	20
	%	0.0	0.0	100.0	0.0	0.0	100.0	0.0	0.0	100.0
Ertapenem	*n*	0	14	56	2	6	58	4	8	8
	%	0.0	20.0	80.0	3.0	9.1	87.9	20.0	40.0	40.0
Gentamicin	*n*	0	0	70	0	0	66	0	0	20
	%	0.0	0.0	100.0	0.0	0.0	100.0	0.0	0.0	100.0
Imipenem	*n*	0	0	70	0	0	66	0	2	18
	%	0.0	0.0	100.0	0.0	0.0	100.0	0.0	10.0	90.0
Levofloxacin	*n*	0	0	70	0	0	66	0	0	20
	%	0.0	0.0	100.0	0.0	0.0	100.0	0.0	0.0	100.0
Meropenem	*n*	0	6	64	0	4	62	0	4	16
	%	0.0	8.6	91.4	0.0	6.1	93.9	0.0	20.0	80.0
Moxifloxacin	*n*	0	0	70	0	0	66	0	0	20
	%	0.0	0.0	100.0	0.0	0.0	100.0	0.0	0.0	100.0
Nitrofurantoin	*n*	0	0	70	0	0	66	0	0	20
	%	0.0	0.0	100.0	0.0	0.0	100.0	0.0	0.0	100.0
Norfloxacin	*n*	0	0	70	0	0	66	0	0	20
	%	0.0	0.0	100.0	0.0	0.0	100.0	0.0	0.0	100.0
Piperacillin/Tazobactam	*n*	0	0	70	0	0	66	0	0	20
	%	0.0	0.0	100.0	0.0	0.0	100.0	0.0	0.0	100.0
Tigecycline	*n*	0	18	52	0	6	60	0	6	14
	%	0.0	25.7	74.3	0.0	9.1	90.9	0.0	30.0	70.0
Tobramycin	*n*	0	0	70	0	0	66	0	0	20
	%	0.0	0.0	100.0	0.0	0.0	100.0	0.0	0.0	100.0
Trimethoprim/Sulfamethoxazole	*n*	12	2	56	8	0	58	4	0	16
	%	17.1	2.9	80.0	12.1	0.0	87.9	20.0	0.0	80.0

R: Resistant. I: Intermediate. S: Sensitive. * The breakpoints are defined by the Clinical and Laboratory Standards Institute (CLSI) [23]. The control *E. coli* strain (ATCC10536) and *K. pneumoniae* strain (ATCC10031) were sensitive to the tested antibiotics.

**Table 4:** Antibiotic resistance/susceptibility patterns of Gram-positive isolates (*n* = 66) by the MicroScan Microbiology System. Data shown are frequencies; *n* (%).

Antibiotics *		*S. epidermidis* *n* = 34 (51.52%)			MRSA *n* = 18 (27.27%)			MSSA *n* = 14 (21.21%)		
		R	I	S	R	I	S	R	I	S
Amikacin	*n*	0	0	34	0	0	18	0	0	14
	%	0.0	0.0	100.0	0.0	0.0	100.0	0.0	0.0	100.0
Amoxicillin/K Clavulanate	*n*	0	0	34	8	10	0	0	0	14
	%	0.0	0.0	100.0	44.44	55.56	0.0	0.0	0.0	100.0
Cefoxitin Screen	*n*	20	7	7	18	0	0	0	0	14
	%	58.8	20.6	20.6	100.0	0.0	0.0	0.0	0.0	100.0
Ciprofloxacin	*n*	0	1	33	8	10	18	2	1	11
	%	0.0	2.90	97.10	44.44	55.56	100.0	14.29	7.14	78.57
Clindamycin	*n*	0	0	34	4	4	10	2	2	10
	%	0.0	0.0	100.0	22.22	22.22	55.56	14.29	14.29	71.42
Daptomycin	*n*.	0	0	34	0	0	18	0	0	14
	%	0.0	0.0	100.0	0.0	0.0	100.0	0.0	0.0	100.0
Erythromycin	*n*	0	3	31	14	4	0	4	5	5
	%	0.0	8.82	91.18	77.78	22.22	0.0	28.58	35.71	35.71
Fosfomycin	*n*	0	0	34	0	0	18	0	0	14
	%	0.0	0.0	100.0	0.0	0.0	100.0	0.0	0.0	100.0
Fusidic Acid	*n*	0	0	34	0	9	9	0	0	14
	%	0.0	0.0	100.0	0.0	50.00	50.00	0.0	0.0	100.0
Gentamicin	*n*	0	3	31	12	6	0	0	0	14
	%	0.0	8.82	91.18	66.67	33.33	0.0	0.0	0.0	100.0
Levofloxacin	*n*	0	0	34	11	7	0	3	1	10
	%	0.0	0.0	100.0	61.11	38.89	0.0	21.43	7.14	71.42
Linezolid	*n*	0	0	34	0	0	18	0	0	14
	%	0.0	0.0	100.0	0.0	0.0	100.0	0.0	0.0	100.0
Mupirocin	*n*	0	0	34	0	9	9	0	0	14
	%	0.0	0.0	100.0	0.0	50.00	50.00	0.0	0.0	100.0
Nitrofurantoin	*n*	0	0	34	0	0	18	0	0	14
	%	0.0	0.0	100.0	0.0	0.0	100.0	0.0	0.0	100.0
Oxacillin	*n*	27	4	3	18	0	0	0	0	14
	%	79.4	11.8	8.8	100.0	0.0	0.0	0.0	0.0	100.0
Penicillin	*n*	20	7	7	18	0	0	0	8	6
	%	58.8	20.6	20.6	100.0	0.0	0.0	0.0	57.14	42.86
Rifampin	*n*	0	0	34	0	0	18	0	0	14
	%	0.0	0.0	100.0	0.0	0.0	100.0	0.0	0.0	100.0
Teicoplanin	*n*	0	0	34	0	0	18	0	0	14
	%	0.0	0.0	100.0	0.0	0.0	100.0	0.0	0.0	100.0
Tetracycline	*n*	0	5	29	2	9	7	0	0	14
	%	0.0	14.71	85.29	11.11	50.00	38.89	0.0	0.0	100.0
Tobramycin	*n*	0	0	34	0	0	18	0	0	14
	%	0.0	0.0	100.0	0.0	0.0	100.0	0.0	0.0	100.0
Trimethoprim/Sulfamethoxazole	*n*	0	0	34	9	9	0	3	3	8
	%	0.0	0.0	100.0	50.00	50.00	0.0	21.43	21.43	57.14
Vancomycin	*n*	0	0	34	0	0	18	0	0	14
	%	0.0	0.0	100.0	0.0	0.0	100.0	0.0	0.0	100.0

R: Resistant. I: Intermediate. S: Sensitive. MRSA: methicillin-resistant *Staphylococcus aureus*. MSSA and methicillin-sensitive *Staphylococcus aureus*. *S. epidermidis*: *Staphylococcus epidermidis*. * The breakpoints are defined by the Clinical and Laboratory Standards Institute (CLSI) [23]. The control *S. aureus* strain (ATCC25923) was sensitive to the tested antibiotics.

## Data Availability

All data are available in the manuscript.
